# Examining the Effects of Sodium Ions on the Binding of Antagonists to Dopamine D_2_ and D_3_ Receptors

**DOI:** 10.1371/journal.pone.0158808

**Published:** 2016-07-05

**Authors:** Claire L. Newton, Martyn D. Wood, Philip G. Strange

**Affiliations:** 1 School of Pharmacy, University of Reading, Reading, RG6 6AJ, United Kingdom; 2 Psychiatry CEDD, GlaxoSmithKline, Harlow, Essex, CM19 5AW, United Kingdom; University of Parma, ITALY

## Abstract

Many G protein-coupled receptors have been shown to be sensitive to the presence of sodium ions (Na^+^). Using radioligand competition binding assays, we have examined and compared the effects of sodium ions on the binding affinities of a number of structurally diverse ligands at human dopamine D_2_ and dopamine D_3_ receptor subtypes, which are important therapeutic targets for the treatment of psychotic disorders. At both receptors, the binding affinities of the antagonists/inverse agonists SB-277011-A, L,741,626, GR 103691 and U 99194 were higher in the presence of sodium ions compared to those measured in the presence of the organic cation, *N*-methyl-D-glucamine, used to control for ionic strength. Conversely, the affinities of spiperone and (+)-butaclamol were unaffected by the presence of sodium ions. Interestingly, the binding of the antagonist/inverse agonist clozapine was affected by changes in ionic strength of the buffer used rather than the presence of specific cations. Similar sensitivities to sodium ions were seen at both receptors, suggesting parallel effects of sodium ion interactions on receptor conformation. However, no clear correlation between ligand characteristics, such as subtype selectivity, and sodium ion sensitivity were observed. Therefore, the properties which determine this sensitivity remain unclear. However these findings do highlight the importance of careful consideration of assay buffer composition for *in vitro* assays and when comparing data from different studies, and may indicate a further level of control for ligand binding *in vivo*.

## Introduction

G protein-coupled receptors (GPCRs) are a large family of seven transmembrane domain cell surface receptors responsible for regulating most biological processes. They present effective drug targets with >40% of marketed therapeutics targeting them, and many drug discovery programs aimed at developing new GPCR-targeting drugs [1,2]. The neurotransmitter dopamine elicits its effects through a family of five GPCRs (dopamine D_1_-D_5_ receptors) which are important targets for the treatment of a number of central nervous system disorders. In particular, the dopamine D_2_ subtype is a major site of action of antipsychotic drugs used to treat disorders such as schizophrenia [3–5]. Many antipsychotic drugs also interact with dopamine D_3_ receptors but the clinical relevance of these interactions remains controversial. However, D_3_ antagonists do show particular promise in the treatment of addiction [6].

A number of GPCRs exhibit sensitivity to the presence of sodium ions (Na^+^) including α_2_-adrenoceptors [7], adenosine A_1_ receptors [8], opiate receptors [9], and dopamine receptors [10–17]. Some agonists display reduced affinity in the presence of sodium ions, and some antagonists increased affinity, while the binding of other ligands is unaffected.

Computational modelling and mutational analysis of several GPCRs have suggested the presence of a sodium binding pocket where sodium ions occupy a site at the centre of a square-pyramidal network of hydrogen bonds formed by Asp^2.50^, Ser^3.39^ and Asn^7.45^ and Ser^7.46^ (residue numbering method of [18]) [19–22]. Asp^2.50^ within TM2 is very highly conserved among GPCRs and has been shown to be pivotal in the regulation of their sensitivity to sodium ions. It is believed to interact with the positively charged sodium ions through electrostatic interactions via its negatively charged carboxylic group [7,10,12,20,21]. Binding of sodium ions within the pocket is believed to cause a conformational change which allosterically modulates ligand binding at the orthosteric site [11]. Interestingly, it has been proposed that this interaction causes a conformational change in the residue Trp^6.48^ so that it mirrors that seen in the crystal structure of the partially inactive form of the receptor [22], suggesting that sodium ion sensitivities of different ligands may reflect their affinity for this inactive conformation. Computational analyses have also suggested that there is an activation-related collapse of the sodium pocket, implicating a specific role of sodium in the signal transduction mechanism [23].

Herein we have studied the effects of sodium ions on the binding of range of antagonists/inverse agonists to dopamine D_2_ and dopamine D_3_ receptors. Whether the two receptor subtypes are differentially regulated by these ions could have important ramifications for future drug development. In order to interrogate the effects of sodium ions using compounds with different receptor binding properties/interactions, the ligands tested were inclusive of different chemical classes (S1 Fig) and receptor subtype selectivity. We postulated that differences seen may help elucidate why different compounds are sensitive/insensitive to the presence of sodium ions.

## Materials and Methods

### Materials

[^3^H]Spiperone (15–30 Ci/mmol) (5 pM—3.5 nM) was obtained from GE Healthcare. Spiperone hydrochloride (spiperone), (+)-butaclamol hydrochloride ((+)-butaclamol), (S)-(−)-sulpiride (sulpiride) and clozapine were obtained from Sigma Aldrich Company Ltd. GR 103691, U 99194 maleate (U 99194) and L-741,626 were obtained from Tocris Cookson Ltd. SB-277011-A was a generous gift from M. Wood, Psychiatry CEDD, GlaxoSmithKline, Harlow, UK.

### Construction of recombinant baculoviruses and BacMam viruses

pCMV5-hD_3_ plasmid DNA was a generous gift from M. Caron, Duke University Medical Centre, Durham, NC, USA. The dopamine D_3_ receptor sequence was amplified by PCR and sub-cloned into the pGEM-T Easy vector (Promega, Southampton, UK) from which it was digested with *Not*I and sub-cloned into the baculovirus transfer vector, pVL1392 (BD Biosciences, Oxford, UK). The resultant pVL1392-hD_3_ plasmid was co-transfected with Baculogold^™^ viral DNA (BD Biosciences) in *Sf*9 insect cells, and underwent homologous recombination to produce recombinant baculoviruses encoding the human dopamine D_3_ receptor (D_3_). Recombinant viruses were isolated and purified using plaque assay purification and were amplified by serial infection of *Sf*9 cells.

Baculoviruses encoding human dopamine D_2S_ receptor with an N-terminal FLAG epitope tag (D_2_) were provided by S. Nickolls, The University of Reading. BacMam viruses encoding wild-type and D80A mutant D_2S_ receptors with N-terminal FLAG epitope tags (D_2_ and D_2_^D80A^, respectively) and wild-type and D75A mutant D_3_ receptors with N-terminal c myc epitope tags (D_3_ and D_3_^D75A^, respectively) were provided by J. Fornwald, GlaxoSmithKline.

### Cell culture

*Sf*9 insect cells were cultured, in suspension, in SF-900 II SFM containing L-glutamine (Invitrogen) supplemented with penicillin (100U/ml) / streptomycin (100 μg/ml). Cells were incubated at 28°C on an orbital shaker (125 rpm) and were sub-cultured every two to three days to maintain a density of 500,000–5,000,000 cells/ml. Cells were infected with baculovirus (multiplicity of infection 5–7) at log-phase growth (~1,000,000 cell/ml) and cells collected after 48 hours. U-2OS cells were cultured, in D-MEM/F-12 (1:1) medium (Invitrogen) supplemented with FBS (10%) and L-glutamine (2 mM). Cells were incubated at 37°C in a humidified atmosphere of 5% CO_2_ and were sub-cultured every three days. Cells were infected with BacMam viruses (multiplicity of infection 25) at log-phase growth (75% confluence) in media supplemented with sodium butyrate (5 mM) and cells collected after 24 hours.

### Membrane preparation

U-2 OS cells were detached from flasks by incubation with Versene (Invitrogen) after washing with phosphate buffered saline (PBS). U-2 OS and *Sf9* cells were collected by centrifugation (200*g*, 10 min) and re-suspended in buffer 1 (20 mM HEPES, 1 mM EDTA, 1 mM EGTA, pH 7.4, 4°C). Cells were then ruptured using an Ultra Turrax^®^ T25 homogenizer (24,000 min^-1^, 4 x 5s) and cell debris removed by centrifugation (400*g*, 5 min, 4°C). The resulting supernatant was collected and centrifuged (47,800*g*, 60 min, 4°C) to collect cell membranes. Membrane pellets were resuspended in buffer 1 using an Ultra Turrax^®^ T25 homogenizer (6,500 min^-1^, 2 x 5s). The protein concentration of the membrane preparations was determined using the Lowry method of protein determination [24] with BSA as a reference standard.

### Radioligand binding assays

#### Radioligand saturation binding assays

Membrane proteins prepared from *Sf*9 cells expressing dopamine D_2_ (25 μg) or dopamine D_3_ receptors (10 μg) were incubated with a range of concentrations of [^3^H]spiperone in buffer 2 (20 mM HEPES, 6 mM MgCl_2_, 1 mM EDTA, 1 mM EGTA, 0.1% BSA, pH 7.4) supplemented, where appropriate, with 100 mM NaCl or 100 mM *N*-methyl-D-glucamine (NMDG). Reactions were performed, in triplicate, in 4 ml LP4 test tubes (1 ml final volume) and were initiated by addition of membrane proteins. Non-specific binding was determined in the presence of (+)-butaclamol (3 μM). Reactions were incubated for 3 hours at 25°C and were terminated by rapid filtration through Whatman glass microfibre GF/C filters using a Brandel cell harvester. After four 3 ml washes with PBS (4°C) filter discs were transferred to scintillation vials and soaked in 2ml Ultima Gold^™^ XR scintillation fluid (Perkin Elmer) for at least 6 hours prior to their radioactivity being determined by liquid scintillation spectrometry. Specific binding was calculated by subtraction of non-specific binding and free radioligand concentration, corrected for ligand depletion calculated. Data were analysed using Prism (Graphpad) and were fitted to hyperbolic equations describing a one-binding site model.

#### Radioligand competition binding assays

Membrane protein prepared from U-2 OS cells (2 μg) or *Sf9* cells (25 μg [D_2_] or 10 μg [D_3_]) expressing wild-type or mutant receptors was incubated with a fixed concentration of [^3^H]spiperone (0.25 nM [D_2_] or 1 nM [D_3_]) and a range of concentrations of competing ligand in buffer 2 (20 mM HEPES, 6 mM MgCl_2_, 1 mM EDTA, 1 mM EGTA, 0.1% BSA, pH 7.4) supplemented with dithiothreitol (0.1 nM) and, where appropriate, 100 mM NaCl or 100 mM NMDG. Non-specific binding was defined using (+)-butaclamol (3 μM) in place of the competing ligand. Reactions were performed, in triplicate, in 4 ml LP4 test tubes (1 ml final volume) [*Sf9*] or deep-well 96-well plates (400μl final volume) [U-2 OS]. Non-specific binding was determined in the presence of (+)-butaclamol (3 μM) and total binding determined in the absence of competing ligand. Reactions were initiated, incubated, terminated and radioactivity measured as described under ‘Radioligand saturation binding assays’. Data are presented as percentage of total binding, after subtraction of non-specific binding. Data were analysed using Prism (Graphpad) and were fitted to sigmoidal equations describing a one-binding site model. Where % inhibition was <100%, parameters were calculated by data extrapolation.

#### Data analysis

Statistical significance of differences between binding parameters were calculated by one-way ANOVA followed by Tukey’s post-hoc test, with a value of p<0.05 considered significant. Before statistical analysis IC_50_ and, K_d_ values were converted to their respective normally distributed negative logarithms (pIC_50_ and pK_d_).

## Results

### The presence of sodium ions does not affect binding of [^3^H]spiperone to dopamine D_2_ or dopamine D_3_ receptors

In order to determine the effect of sodium ions on the binding of ligands to the dopamine D_2_ and dopamine D_3_ receptors, it was first necessary to select a suitable radioligand which interacted with both receptors at high affinity and the binding of which was not itself affected by the presence of these ions.

Saturation radioligand binding assays were performed using [^3^H]spiperone, in the presence of 100 mM NaCl or in the presence of 100 mM NMDG, an organic cation, used to control for ionic strength (Table 1). At both receptors, [^3^H]spiperone affinity was not significantly different in either buffer condition. Complete removal of monovalent cations from the buffer also had no effect on the measured affinity of [^3^H]spiperone at either receptor (p>0.05). Although the B_max_ measured in the presence of NMDG was higher than in the presence of Na+ or absence of monovalent cations, the choice of buffer composition did not significantly alter receptor expression levels measured for either receptor (p>0.05). The lack of sodium sensitivity and the sub-nanomolar affinity of [^3^H]spiperone for both receptor subtypes therefore makes it a suitable choice of radioligand for this study.

**Table 1:** Saturation analyses of [^3^H]spiperone binding in the presence or absence of monovalent cations.

	D_2_		D_3_	
	B_max_ (pmol/mg)	pK_d_^a^	B_max_ (pmol/mg)	pK_d_^a^
	0.60 ± 0.14	10.09 ± 0.12 (0.08 nM)	4.21 ± 0.64	9.62 ± 0.10 (0.24 nM)
+ NaCl	0.65 ± 0.10	9.92 ± 0.08 (0.12 nM)	5.39 ± 0.43	9.49 ± 0.11 (0.32 nM)
+ NMDG	1.26 ± 0.49	9.76 ± 0.10 (0.17 nM)	4.57 ± 0.48	9.46 ± 0.03 (0.35 nM)

Radioligand saturation binding analyses of [^3^H]spiperone, to membranes prepared from Sf9 cells expressing D_2_ or D_3_ receptors, were measured in the absence of monovalent cations, in the presence of 100 mM NaCl or in the presence of 100 mM NMDG. Data are mean ± SEM from 4 independent experiments.

^a^Values are presented as their negative logarithms (pK_d_). Corresponding K_d_ concentrations are shown in parentheses.

### Mutation of Asp^2.50^ in dopamine D_2_ and dopamine D_3_ receptors abolishes their sodium ion sensitivity

It has been previously demonstrated that Asp^2.50^ is critical to the sodium ion sensitivity of many GPCRs, including dopamine D_2_ receptors, in which mutation of this residue (D80) to alanine abolishes sodium ion effects [12]. In order to confirm whether mutation of the corresponding residue in the dopamine D_3_ receptor (D75) also ablates its sodium ion sensitivity, mutant receptors in which these aspartic acid residues are replaced with alanine were produced. Sodium sensitivity of the binding of a substituted benzamide (sulpiride) was then examined. The binding of this class of dopamine receptor antagonists has previously been demonstrated to be strongly modulated in the presence of sodium ions [12].

**Fig 1:**
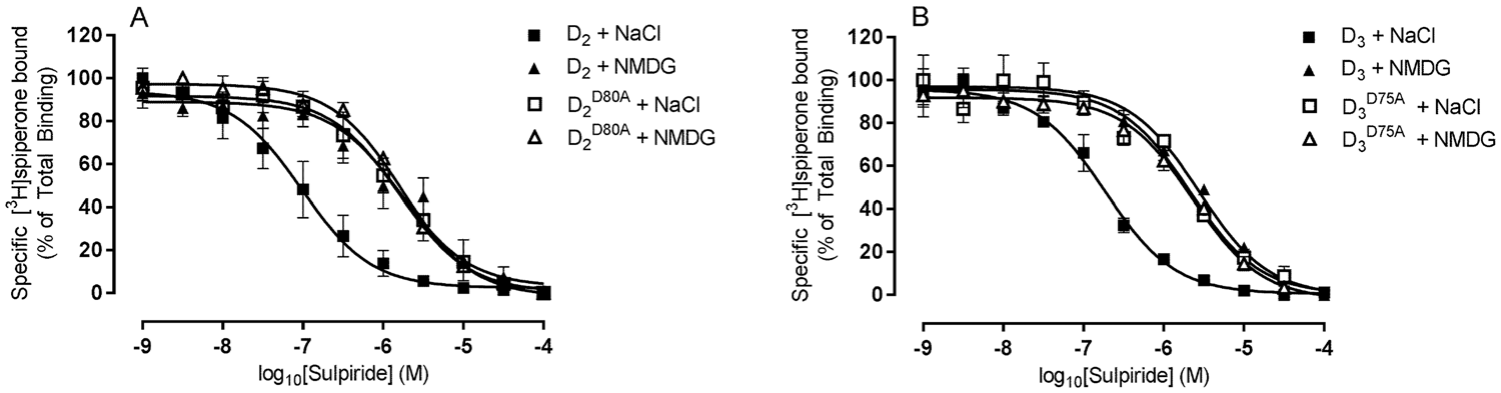
Sodium ions modulate the binding of sulpride to wild-type dopamine D_2_ and D_3_ receptors but not to mutant dopamine D_2_^D80A^ and D_3_^D75A^ receptors. Competition analyses of the inhibition of [^3^H]spiperone binding to membranes prepared from U-2 OS cells expressing (A) wild-type dopamine D_2_ receptors (filled symbols)/dopamine D_2_^D80A^ mutant receptors (open symbols) or (B) wild-type dopamine D_3_ receptors (filled symbols)/dopamine D_3_^D75A^ mutant receptors (open symbols), by sulpiride, were performed in the presence of 100 mM NaCl (■/□) or 100 mM NMDG (▲/Δ). Data are mean ± SEM of at least 3 independent experiments.

The sodium ion sensitivity of the binding of sulpiride to wild-type and mutant receptors was investigated by performing radioligand competition binding assays with [^3^H]spiperone in the presence of NMDG or NaCl (Fig 1). While the affinity of sulpiride binding to the wild-type dopamine D_2_ (pIC_50_ [NaCl] = 7.02 ± 0.25; pIC_50_ [NMDG] = 5.78 ± 0.19) and D_3_ receptors (pIC_50_ [NaCl] = 6.74 ± 0.13; pIC_50_ [NMDG] = 5.57 ± 0.03) was significantly increased (15 to 17-fold; p<0.001) in the presence of NaCl, there was no significant difference in affinity measured in the two buffer conditions at the dopamine D_2_^D80A^ (pIC_50_ [NaCl] = 5.85 ± 0.21; pIC_50_ [NMDG] = 5.76 ± 0.08) or dopamine D_3_^D75A^ (pIC_50_ [NaCl] = 5.70 ± 0.15; pIC_50_ [NMDG] = 5.67 ± 0.14) mutant receptors (p>0.05). At the dopamine D_2_^D80A^ and D_3_^D75A^ mutant receptors, the affinity of sulpride in either buffer condition reflected that measured for the corresponding wild-type receptor in the presence of NMDG.

### The presence of sodium ions affects the binding of several antagonists to dopamine D_2_ and dopamine D_3_ receptors

**Fig 2:**
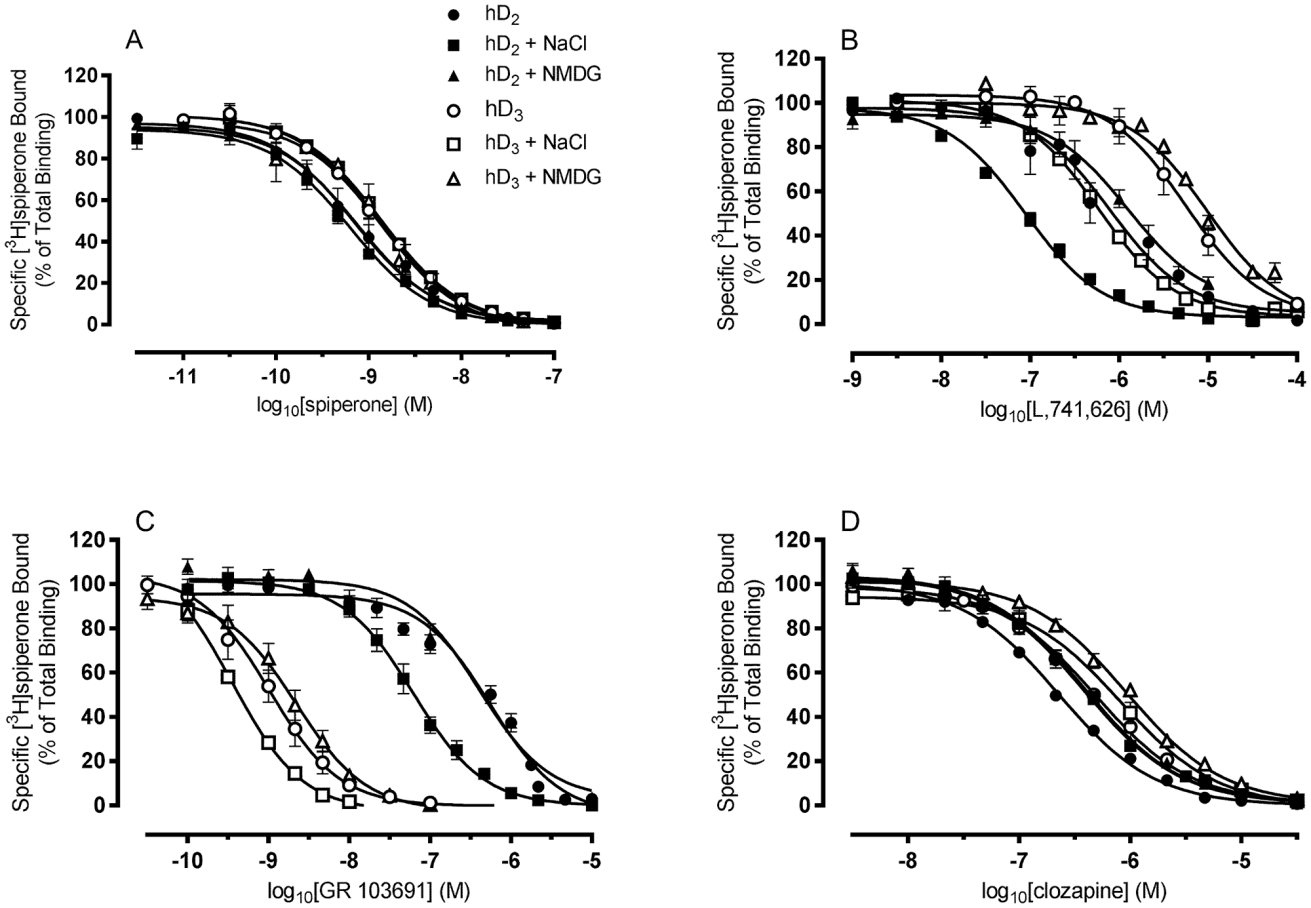
Binding affinities of several inverse agonists/antagonists are affected by the presence of sodium ions. Competition analyses of the inhibition of [^3^H]spiperone binding to membranes prepared from Sf9 cells expressing dopamine D_2_ (filled symbols) or dopamine D_3_ receptors (open symbols), by (A) spiperone, (B) L,741,626, (C) GR 103691 and (D) clozapine, were performed in the absence of monovalent cations (●/○), in the presence of 100 mM NaCl (■/□) or 100 mM NMDG (▲/Δ). Data are mean ± SEM of at least 3 independent experiments.

The effect of the presence of sodium ions on the binding of a range of antagonists/inverse agonists to the two receptors was then determined (Fig 2 and Table 2). At both receptors, the binding affinities of spiperone and (+)-butaclamol were not significantly different in buffers containing NaCl or NMDG (p>0.05), suggesting that the binding of these ligands is unaffected by the presence of sodium ions (Fig 2A and Table 2). Conversely, at both receptors, SB-277011-A, U 99194, GR 103691 and L,741,626 (Fig 2B, 2C and Table 2) all exhibited significantly higher affinities in buffer containing NaCl compared to that containing NMDG. As affinities measured in the presence of NMDG were not significantly different from those measured in the absence of any monovalent cations, these effects appear to be specific to the presence of sodium ions, rather than to changes in ionic strength.

**Table 2:** Effects of sodium ions and NMDG on the affinity of various antagonists/inverse agonists.

	D_2_			D_3_		
		+ NaCl	+ NMDG		+ NaCl	+ NMDG
SB-277011-A	4.78 ± 0.12(16600 nM)	5.25 ± 0.05^b^(5660 nM)	4.53 ± 0.05(29800 nM)	6.83 ± 0.13(150 nM)	7.42 ± 0.03^b^(38.3 nM)	6.64 ± 0.03(231 nM)
U 99194	4.25 ± 0.13(55700 nM)	5.02 ± 0.07^b^(9480 nM)	4.14 ± 0.18(72900 nM)	5.30 ± 0.18(5050 nM)	6.27 ± 0.06^b^(532 nM)	5.28 ± 0.08(5200 nM)
GR 103691	6.45 ± 0.13(354 nM)	7.22 ± 0.07^b^(60.3nM)	6.27 ± 0.02(543 nM)	8.97 ± 0.10(1.08 nM)	9.34 ± 0.04^b^(0.46 nM)	8.64 ± 0.08(2.29 nM)
Spiperone	9.16 ± 0.17(0.70 nM)	9.21 ± 0.05(0.62 nM)	9.14 ± 0.06(0.73 nM)	8.93 ± 0.07(1.18 nM)	8.91 ± 0.03(1.24 nM)	8.72 ± 0.18(1.91 nM)
(+)-butaclamol	8.64 ± 0.04(2.31 nM)	8.53 ± 0.11(2.97 nM)	8.62 ± 0.28(2.38 nM)	7.88 ± 0.12(13.3 nM)	7.82 ± 0.09(15.0 nM)	7.78 ± 0.03(16.8 nM)
L,741,626	5.92 ± 0.14(1200 nM)	7.07 ± 0.07^b^(86.1 nM)	5.68 ± 0.09(2110 nM)	5.21 ± 0.21(6120 nM)	6.23 ± 0.01^b^(589 nM)	5.00 ± 0.11(9930 nM)
Clozapine	6.64 ± 0.02^b^(228 nM)	6.38 ± 0.06(413 nM)	6.40 ± 0.03(395 nM)	6.30 ± 0.03^b^(500 nM)	6.17 ± 0.11(681 nM)	6.04 ± 0.03(918 nM)

Competition analyses of the inhibition of [^3^H]spiperone binding, to membranes prepared from Sf9 cells expressing D_2_ or D_3_ receptors, by various ligands, were performed, in the absence of monovalent cations, in the presence of 100 mM NaCl or in the presence of 100 mM NMDG. Data are mean ± SEM of at least 3 independent experiments. Values are presented as their negative logarithms (pIC_50_). Corresponding IC_50_ concentrations are shown in parentheses.

^b^p< 0.05 (one-way ANOVA, followed by Tukey’s post-hoc test) for comparison with buffer containing NMDG.

At both receptors, those compounds which exhibited sodium ion sensitivity displayed varying levels of sodium ion-modulated differences in affinity. These varied from 17-25-fold for L,741,626 to 5-6-fold, for SB-277011-A (comparing affinities measured in the presence of NaCl and NMDG). Additionally, although SB-277011-A displayed quantitatively similar sodium ion-modulated increases in affinity (5-6-fold) at both receptors, the increase in affinity of GR 103691 and L,741,626 at the D_3_ receptor was more modest than that observed at the D_2_ receptor, while the increase in affinity of U 99194 was more modest at the D_2_ receptor (Table 2). These differences had some effect on the observed rank order of affinity of the compounds at each receptor measured in the different buffer conditions. An example is, clozapine which displayed similar affinity to GR 103691 at the dopamine D_2_ receptor in the presence of NMDG (p>0.05) but significantly lower affinity (7-fold) than GR 103691 in the presence of NaCl (p<0.05) (Table 2).

Interestingly, and in contrast to the sodium ion-modulated effects described above, at both receptors the affinity of clozapine was similar in the presence of both NaCl and NMDG and moderately increased (2-fold) upon removal of monovalent cations from the buffer, suggesting an effect of ionic strength rather than the presence of specific cations on the binding of this ligand to these receptors (Fig 2D and Table 2).

## Discussion

We have examined sodium ion sensitivity of the binding of a range of ligands to dopamine D_2_ and dopamine D_3_ receptors. Although prior studies have described the effects of sodium ions on the binding of ligands to these receptors, the panel of ligands examined, particularly with respect to the D_3_ receptor, has been extremely limited [17,25,26]. It was our intention to determine whether there was any differential regulation of ligand binding to the two receptor subtypes by sodium ions and if so, whether this was impacted by ligand structure and/or subtype selectivity. Thus, in the present study, a selection of ligands covering different chemical/therapeutic classes and with different selectivity for the two dopamine receptor subtypes were examined (S1 Fig). In addition, comparisons were made between buffer conditions containing sodium ions (NaCl), NMDG as a control for ionic strength, or in the absence of monovalent cations in order to elucidate sodium ion or ionic strength-specific effects.

The binding affinities of some ligands ((+)-butaclamol and spiperone) were unaffected by the presence of sodium ions at either receptor. Although sodium sensitivity of the binding of (+)-butaclamol to the D_3_ receptor has not previously been studied, our observations that the binding of spiperone to both the D_2_ and D_3_ receptors and (+)-butaclamol to the D_2_ receptor is not sensitive to the presence of sodium ions are in agreement with previous studies [14,27–29]. Our finding that the number of D_2_ receptor binding sites labelled by the radioligand [^3^H]-spiperone is not affected by the presence/absence of sodium ions is also corroborated by a previous study reporting no effect of these ions on B_max_ values in saturation binding experiments performed using mammalian cells expressing D_2_ receptors [14].

The binding affinities of other ligands were increased, at both receptors, in the presence of sodium ions (SB-277011-A, U 99194, GR 103691 and L,741,626). Each of these ligands exhibited differing degrees of sensitivity, with the sodium-induced increases in affinity varying from 5-6-fold (SB-277,011-A) to 17-25-fold (L,741,616). The sodium ion sensitivities of binding of these ligands to neither the D_2_ nor D_3_ receptors have previously been reported. However previous studies have demonstrated that the affinity of 1,4-disubstituted aromatic piperidine/piperazine compounds (1,4-DAPs) for D_2_ receptors is increased in the presence of sodium ions and that this can be attributed to an enhancement of binding pocket accessibility upon sodium binding [11]. Compounds L,741,626 and GR 103691, utilised in the present study, are both 1,4–DAPs. Therefore, the observed increased in affinity in the presence of sodium ions is unsurprising. However, it is interesting that the binding of spiperone, a 1,4-DAP analogue with a spirocyclic lactam attached, is not sensitive to the presence of sodium, while the binding of U 99194, a compound of unrelated structure, does display sodium sensitivity.

Comparisons were made between affinities measured in the presence of NaCl or the organic cation, NMDG, and indicated that that, in the majority of cases, the effects seen were specific to the presence of sodium ions and not due to changes in buffer ionic strength, supporting the theory that many GPCRs contain sodium ion-specific binding pockets. However, it is interesting to note that, although not significantly different, for those ligands which displayed sodium ions sensitivities the affinities measured in the presence of NMDG were consistently lower than those measured in the absence of monovalent cations.

Interestingly, the binding affinity of clozapine was not different when compared in conditions containing sodium or NMDG, while removal of monovalent cations resulted in a significant increase in binding affinity at both receptors. These data are indicative of a sensitivity to the buffer ionic strength (or to the general presence of monovalent cations) rather than the presence of specific cations. However, the effects seen are very modest (only 2-fold) and therefore will have limited implications. These data are in agreement with a previous study which demonstrated that the affinity of clozapine at the D_3_ receptor is not different in the presence of either NaCl or NMDG [17]. However, the data in the present study data contradict those of previous studies in which the affinity of clozapine at the D_2_ receptor was found to differ between buffers containing NaCl and NMDG [27,28]. These previous studies present contradictory findings with respect to the effects of sodium ions on clozapine affinity, with both sodium-induced increases and decreases in affinity being reported. In addition, affinities in the absence of either cation were not measured, so it is not possible to determine whether there were also unspecific cation effects, as demonstrated in the present study. The discrepancies noted may be related to the use of cell background used for the measurements with mammalian cells being utilised in the previous studies as opposed to the *Sf*9 cell system employed here. Furthermore, in some mammalian cell systems, clozapine has been shown to display binding to two distinct affinity states of the D_2_ receptor, possibly reflecting its ability to distinguish between G protein-coupled and free forms. Indeed, Malmberg et al. reported biphasic displacement curves for clozapine binding to D_2_ receptors in their mammalian cell system [17]. In *Sf*9 cells, coupling to endogenous G proteins has been shown to be poor [30]. Therefore, differences in G protein-coupling between these studies may also contribute to the differences seen.

It has been postulated that the interaction of GPCRs with sodium ions causes a conformational change to a form that potentially reflects the partially inactive state of the receptor. Therefore, it would be predicted that the affinities of antagonist / inverse agonist ligands (believed to stabilize the inactive state of the receptor) would be increased in in the presence of sodium ions while agonist affinities would be decreased for the same reason. Indeed, such effects of sodium on agonist/antagonist binding have previously been reported for several GPCRs [8,9,14,31,32]. However, in the present study, we demonstrate that different antagonist/inverse agonist ligands can have differing affinities for the sodium-bound receptor. Although the affinities of many were increased, others were unaffected or, in the case of clozapine, were decreased in the presence of sodium, indicating that sodium ion sensitivity is not related to efficacy.

We found that mutation of the conserved aspartate residue, Asp^2.50^_,_ previously demonstrated to be pivotal for sodium ion regulation of the D_2_ receptor [7,12,20–22], also abolished sodium-induced effects on binding of the substituted benzamide, sulpiride, to the D_3_ receptor suggesting a similar mode of interaction with these ions for the two receptors. Binding to both receptors was also affected by the presence of sodium ions in a similar way, suggesting that sodium ion interaction causes parallel conformational changes in both subtypes. Therefore, it would be predicted that compounds with similar properties and thus similar interactions with the receptors, would be affected in the same way. However, there was no correlation between subtype selectivity of the compounds and their sensitivity to sodium ions as SB-277011A displayed dopamine D_3_ receptor selectivity and L,741,626 dopamine D_2_ receptor selectivity, but the affinities of both were increased in the presence of sodium ions. Furthermore, SB277011-A, a large multi-cyclic carboxamide 1,4,-DAP, displays similar sensitivity to sodium ions as U 99194, a much smaller compound with very little structural similarity. Therefore, it is likely that the sodium sensitive compounds share binding interactions with sites revealed upon sodium binding and which are common to the two receptors, while subtype selectivity of these compounds is determined by interactions with residues not affected by sodium interactions. Indeed, while the binding sites of both receptors share very high homology, molecular modelling studies have indicated differences in the extracellular part of the binding pocket which have been predicted to be involved in conferring compound selectivity for the two receptors [33].

Although the presence of sodium ions affects both receptors in a similar manner, differences were observed in the degree to which their affinities were altered at the two receptors. Again, these differences did not correlate with the subtype selectivity of the compounds, but it is interesting to note that the sodium-induced effects on binding of the two structurally related compounds (GR 103691 and L741,626) were both far more modest at the D_3_ receptor than the D_2_ receptor. Although the specific properties and interactions which determine sodium ion sensitivity of the compounds remain unclear, further interrogation through molecular docking analysis may be able to shed light on these effects. Indeed, a recent study used a combination of binding assays and computational modelling analyses (utilising the published crystal structure of the D_3_ receptor reported by Chien et al. [34]) to study the impact of sodium ion binding on the conformation and ligand binding pocket of D_2_ and D_3_ receptors [29]. They found that the presence of sodium had similar allosteric effects on the ligand binding site of both receptors and removal of the ion caused a weakening/breaking of an important interaction between the side chains of the conserved residues, Asp^3.32^ and Tyr^7.43^, (located between the sodium binding pocket and ligand binding site). This interaction is critical for the binding of the sodium sensitive substituted benzamide ligands, sulpiride and eticlopride. Thus, the binding affinity of these ligands is decreased upon disruption of this bond in the absence of sodium ions, whereas the more extensive receptor binding interactions of the sodium-insensitive compound, spiperone, are though to mask these effects [29]. Although the compounds tested in the present study are structurally unrelated to the substituted benzamide ligands, a similar mechanism may also account for their sodium sensitivities.

Although the presence of sodium ions was found to affect both receptors in a similar manner, some evidence of differential regulation of the two receptor subtypes by sodium ions was observed (with relation to the magnitude of the effects seen), but these differences were minimal. However, this study does highlight that assay buffer composition can have a significant effect on measured ligand affinities. This is an important observation, especially when comparing data from different laboratories and when analysing the pharmacological properties of potential therapeutics. Modulation of ligand binding by sodium ions may also have physiological relevance. For example, in dopaminergic neurons, action potentials result in large transient fluctuations in intra- and extracellular sodium ion concentrations. Therefore, effects on binding of exogenous and endogenous compounds *in vivo* should also be considered.

## Supporting Information

S1 Fig Chemical Structures of compounds tested. (PDF)

S1 File Data File. (XLSX)
