# Supplementary figures and images for: Examining the Effects of Sodium Ions on the Binding of Antagonists to Dopamine D2 and D3 Receptors
Source: PLoS One. 2016 Jul 5;11(7):e0158808. doi: 10.1371/journal.pone.0158808 (PMC4933336; doi:10.1371/journal.pone.0158808)

SB-277011-A

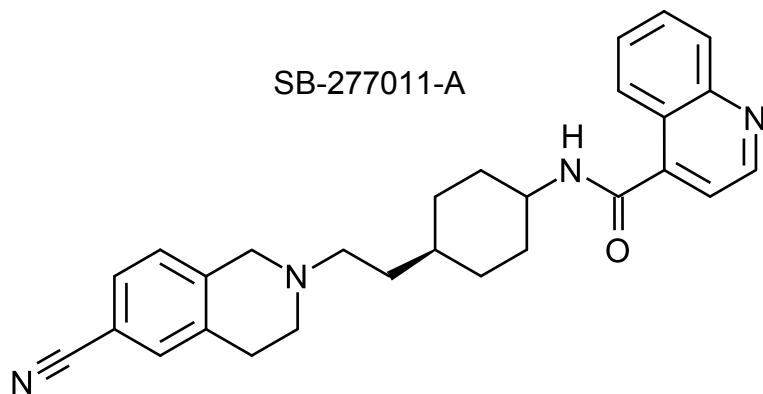

U 99194 maleate

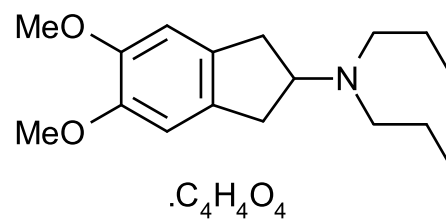

GR 103691

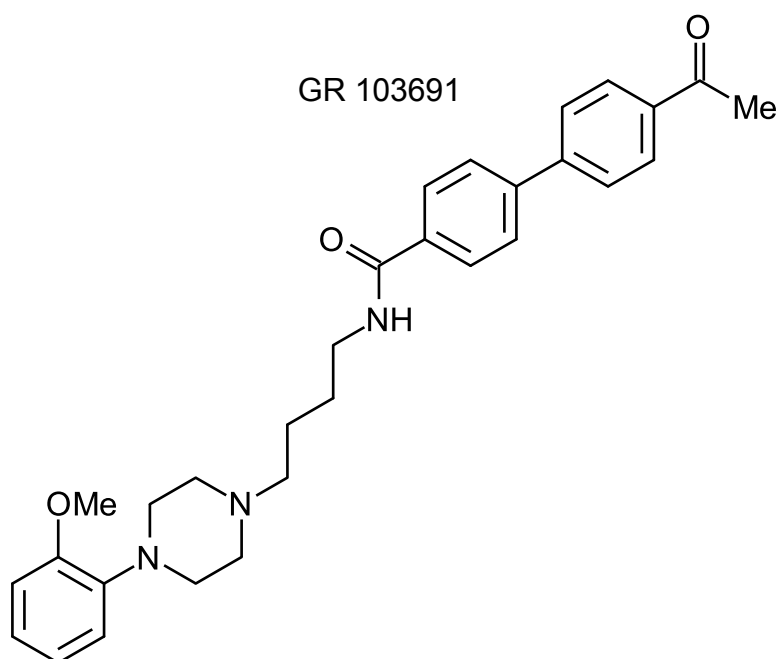

(+)-butaclamol hydrochloride

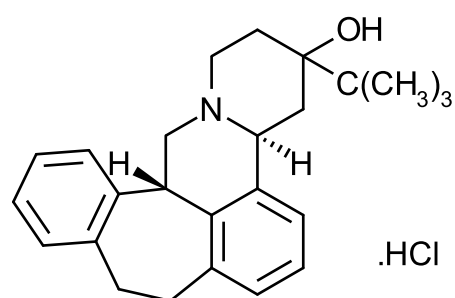

Spiperone hydrochloride

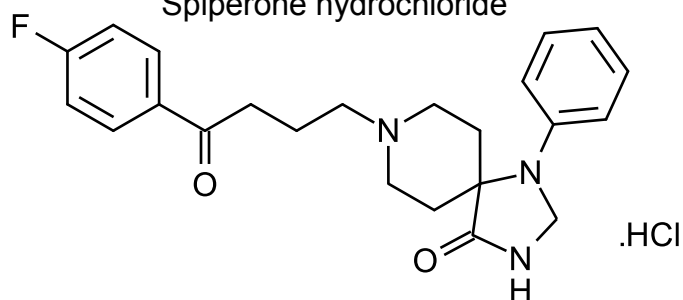

Clozapine

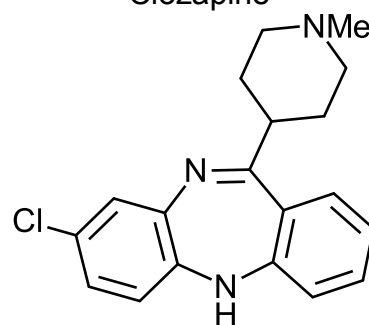

L,741,626

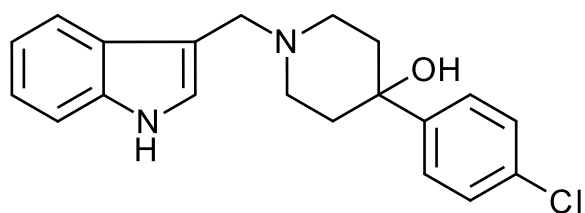

Supplement: S1 Fig — (PDF) [file pone.0158808.s001.pdf]
